# (1S*,3S*,8S*,10S*)-10-Fluoro-15-oxa­tetra­cyclo­[6.6.1.0^1,10^.0^3,8^]penta­deca-5,12-dien-3-ol

**DOI:** 10.1107/S1600536811026857

**Published:** 2011-07-09

**Authors:** Goverdhan Mehta, Saikat Sen

**Affiliations:** a Department of Organic Chemistry, Indian Institute of Science, Bangalore 560 012, Karnataka, India

## Abstract

The title compound, C_14_H_17_FO_2_, was obtained from *anti*-4a,9a:8a,10a-diep­oxy-1,4,4a,5,8,8a,9,9a,10,10a-deca­hydro­anthra­cene *via* tandem hydrogen-fluoride-mediated epoxide ring-opening and transannular oxacyclization. With the two cyclo­hexene rings folded towards the oxygen bridge, the title tetra­cyclic fluoro­alcohol mol­ecule displays a conformation remin­iscent of a pagoda. The crystal packing is effected *via* inter­molecular O—H⋯O hydrogen bonds, which link the mol­ecules into a zigzag chain along the *b* axis.

## Related literature

For applications of organofluorine compounds as pharmaceuticals, see: Kirsch (2004 ▶); Bégué & Bonnet-Delpon (2006 ▶); Müller *et al.* (2007 ▶). For the use of diethyl­amino­sulfur trifluoride, 1-chloro­methyl-4-fluoro­diazo­niabicyclo­[2.2.2]octane bis­(tetra­fluoro­borate) and pyridinium poly(hydrogen fluoride) as reagents for selective introduction of C—F bonds, see: Middleton (1975 ▶); Olah *et al.* (1979 ▶); Banks *et al.* (1992 ▶). For the preparation of the title compound, see: Mehta *et al.* (2007 ▶); Mehta & Sen (2010 ▶).
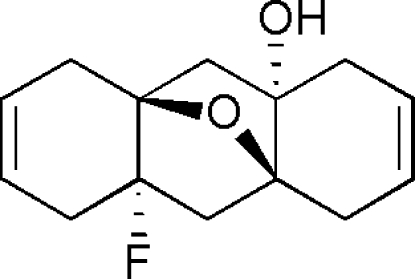


## Experimental

### 

#### Crystal data

C_14_H_17_FO_2_
*M* *_r_* = 236.28
Monoclinic, 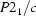

*a* = 8.1603 (6) Å
*b* = 10.9148 (8) Å
*c* = 13.5558 (10) Å
β = 96.285 (3)°
*V* = 1200.13 (15) Å^3^
*Z* = 4
Mo *K*α radiation
μ = 0.10 mm^−1^
*T* = 291 K
0.26 × 0.22 × 0.12 mm

#### Data collection

Bruker SMART APEX CCD area-detector diffractometer
Absorption correction: multi-scan (*SADABS*; Sheldrick, 2003 ▶) *T*_min_ = 0.976, *T*_max_ = 0.989
10752 measured reflections
2454 independent reflections
2038 reflections with *I* > 2σ(*I*)
*R*_int_ = 0.020

#### Refinement

*R*[*F* ^2^ > 2σ(*F* ^2^)] = 0.041
*wR*(*F* ^2^) = 0.113
*S* = 1.03
2454 reflections
155 parameters
H-atom parameters constrained
Δρ_max_ = 0.19 e Å^−3^
Δρ_min_ = −0.21 e Å^−3^

### 

Data collection: *SMART* (Bruker, 1998 ▶); cell refinement: *SAINT* (Bruker, 1998 ▶); data reduction: *SAINT*; program(s) used to solve structure: *SIR92* (Altomare *et al.*, 1994 ▶); program(s) used to refine structure: *SHELXL97* (Sheldrick, 2008 ▶); molecular graphics: *ORTEP-3 for Windows* (Farrugia, 1997 ▶) and *CAMERON* (Watkin *et al.*, 1993 ▶); software used to prepare material for publication: *PLATON* (Spek, 2009 ▶).

## Supplementary Material

Crystal structure: contains datablock(s) global, I. DOI: 10.1107/S1600536811026857/is2740sup1.cif

Structure factors: contains datablock(s) I. DOI: 10.1107/S1600536811026857/is2740Isup2.hkl

Supplementary material file. DOI: 10.1107/S1600536811026857/is2740Isup3.cml

Additional supplementary materials:  crystallographic information; 3D view; checkCIF report

## Figures and Tables

**Table 1:** Hydrogen-bond geometry (Å, °)

*D*—H⋯*A*	*D*—H	H⋯*A*	*D*⋯*A*	*D*—H⋯*A*
O1—H1⋯O2^i^	0.82	2.14	2.9554 (14)	177

Symmetry code: (i) 
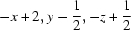
.

**Fig. 1.**
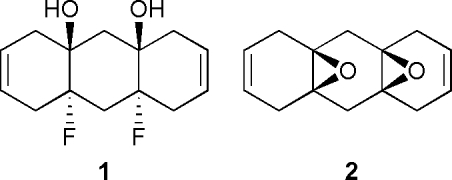
Chemical structural diagrams of 1 and 2.

**Fig. 2.**
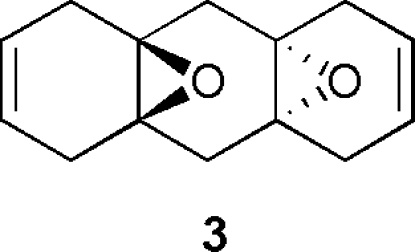
Chemical structural diagram of 3.

**Fig. 3.**
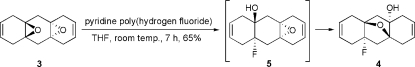
Preparation of the title compound, fluoroalcohol 4, from the anti- diepoxide 3.

**Fig. 4.**
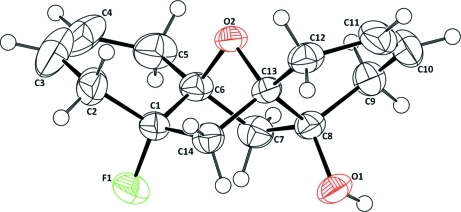
The molecular structure of the title compound 4, with the atom numbering scheme. Displacement ellipsoids for non-H atoms are drawn at the 50% probability level.

**Fig. 5.**
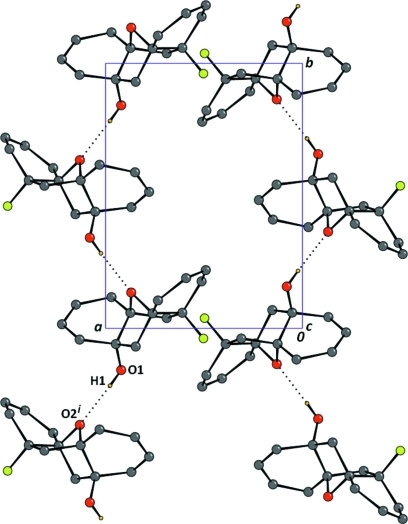
A packing diagram of the title compound 4, viewed along the c axis. Non-interacting hydrogen atoms have been removed for clarity. Dotted lines indicate the O—H···O hydrogen bonds.

## supplementary crystallographic information

### Comment

Organofluorine compounds, while rarely occurring naturally, constitute around 20% of all known pharmaceuticals (Bégué & Bonnet-Delpon, 2006; Müller *et al.*, 2007). The wide- spread applications of fluorinated organic compounds in the therapeutic arena has been attributed to the fact that incorporating fluorine in a drug can significantly enhance its lipophilicity and *in vitro* stability towards cytochrome P450 enzymatic oxidation (Kirsch, 2004; Müller *et al.*, 2007).

Not surprisingly, various reagents, such as diethylaminosulfur trifluoride (DAST) (Middleton, 1975) and 1-chloromethyl-4-fluorodiazoniabicyclo[2.2.2]octane bis(tetrafluoroborate) (Selectfluor) (Banks *et al.*, 1992) have been developed over the years for achieving controlled and selective introduction of C—F bonds. The Olah's reagent [pyridinium poly(hydrogen fluoride)] was, in this context, among the first such fluorinating agents to be reported (Olah *et al.*, 1979; Müller *et al.*, 2007).

In a recent endeavor, we employed this reagent as means of accessing the difluorodiol 1*via* one-pot HF-mediated ring-opening in the *syn*-diepoxide 2 (Fig. 1; Mehta & Sen, 2010). The complete regio- and stereoselectivity, observed in this bis-fluorination step, was intriguing and goaded us to investigate the outcome of reacting pyridine poly(hydrogen fluoride) with the *anti*-diepoxide 3 (Fig. 2; Mehta *et al.*, 2007).

The title compound 4, bearing a 7-oxanorbornane core inscribed in a 1,4,4a,5,8,8a,9,9a,10,10*a*-decahydroanthracene framework, was obtained as the major product. Formation of the tetracyclic fluoroalcohol 4 can be explained by an initial HF-mediated epoxide ring opening in 3 to yield the fluorohydrin 5, followed by a novel variant of a hydroxy-mediated bishomo-Payne rearrangement in 5 to afford 4 (Fig. 3).

The crystal structure of 4 was solved and refined in the centrosymmetric monoclinic space group *P*2_1_/*c* (*Z* = 4). The two flanking cyclohexene rings, folded towards the oxa bridge of the bicyclic core, and the pendant *syn*-4-fluoro-butan-1-ol moiety afforded the molecule an interesting pagoda-like architecture (Fig. 4). Crystal packing in 4 was effected *via* the agency of intermolecular O—H···O hydrogen bonds which linked the tetraacetate molecules into zigzag chains along the *b* axis (Fig. 5).

### Experimental

A solution of the anti-diepoxide 3 (35 mg, 0.162 mmol) in 1 ml of dry THF was treated with pyridine- poly(hydrogen fluoride) (0.5 ml, 27.5 mmol) at 273 K. The reaction was allowed to proceed for 7 h at ambient temperature. The mixture was then quenched with saturated sodium bicarbonate solution. The product was extracted with ethyl acetate; the combined extracts were washed with brine and then dried over anhydrous sodium sulfate. Removal of solvent, column chromatography over silica gel and subsequent recrystallization using 20% EtOAc-petroleum ether afforded 4 (25 mg, 65%) as a colorless crystalline solid.

### Refinement

The methine (CH) and methylene (CH_2_) H atoms were placed in geometrically idealized positions with C—H distances 0.93 and 0.97 Å respectively, and allowed to ride on their parent atoms with *U*_iso_(H) = 1.2*U*_eq_(C). The hydroxyl hydrogen atom was constrained to an ideal geometry with the O—H distance fixed at 0.82 Å and *U*_iso_(H) = 1.5*U*_eq_(O). During refinement, the hydroxyl group was however allowed to rotate freely about its C—O bond.

### Crystal data

**Table:**

C_14_H_17_FO_2_	*F*(000) = 504
*M**_r_* = 236.28	*D*_x_ = 1.308 Mg m^−^^3^
Monoclinic, *P*2_1_/*c*	Mo *K*α radiation, λ = 0.71073 Å
Hall symbol: -P 2ybc	Cell parameters from 4978 reflections
*a* = 8.1603 (6) Å	θ = 2.4–26.4°
*b* = 10.9148 (8) Å	µ = 0.10 mm^−^^1^
*c* = 13.5558 (10) Å	*T* = 291 K
β = 96.285 (3)°	Block, colorless
*V* = 1200.13 (15) Å^3^	0.26 × 0.22 × 0.12 mm
*Z* = 4	

### Data collection

**Table:**

Bruker SMART APEX CCD area-detector diffractometer	2454 independent reflections
Radiation source: fine-focus sealed tube	2038 reflections with *I* > 2σ(*I*)
graphite	*R*_int_ = 0.020
φ and ω scans	θ_max_ = 26.4°, θ_min_ = 2.4°
Absorption correction: multi-scan (*SADABS*; Sheldrick, 2003)	*h* = −10→10
*T*_min_ = 0.976, *T*_max_ = 0.989	*k* = −13→12
10752 measured reflections	*l* = −16→16

### Refinement

**Table:**

Refinement on *F*^2^	Primary atom site location: structure-invariant direct methods
Least-squares matrix: full	Secondary atom site location: difference Fourier map
*R*[*F*^2^ > 2σ(*F*^2^)] = 0.041	Hydrogen site location: inferred from neighbouring sites
*wR*(*F*^2^) = 0.113	H-atom parameters constrained
*S* = 1.03	*w* = 1/[σ^2^(*F*_o_^2^) + (0.0537*P*)^2^ + 0.3336*P*] where *P* = (*F*_o_^2^ + 2*F*_c_^2^)/3
2454 reflections	(Δ/σ)_max_ < 0.001
155 parameters	Δρ_max_ = 0.19 e Å^−^^3^
0 restraints	Δρ_min_ = −0.21 e Å^−^^3^

### Special details

**Table:**

Geometry. All e.s.d.'s (except the e.s.d. in the dihedral angle between two l.s. planes) are estimated using the full covariance matrix. The cell e.s.d.'s are taken into account individually in the estimation of e.s.d.'s in distances, angles and torsion angles; correlations between e.s.d.'s in cell parameters are only used when they are defined by crystal symmetry. An approximate (isotropic) treatment of cell e.s.d.'s is used for estimating e.s.d.'s involving l.s. planes.
Refinement. Refinement of *F*^2^ against ALL reflections. The weighted *R*-factor *wR* and goodness of fit *S* are based on *F*^2^, conventional *R*-factors *R* are based on *F*, with *F* set to zero for negative *F*^2^. The threshold expression of *F*^2^ > σ(*F*^2^) is used only for calculating *R*-factors(gt) *etc*. and is not relevant to the choice of reflections for refinement. *R*-factors based on *F*^2^ are statistically about twice as large as those based on *F*, and *R*- factors based on ALL data will be even larger.

### Fractional atomic coordinates and isotropic or equivalent isotropic displacement parameters (Å^2^)

**Table:**

	*x*	*y*	*z*	*U*_iso_*/*U*_eq_	
F1	0.50293 (10)	−0.03822 (8)	0.17755 (7)	0.0539 (3)	
O1	0.92031 (13)	−0.15772 (9)	0.33199 (8)	0.0491 (3)	
O2	0.86964 (11)	0.13521 (8)	0.21690 (6)	0.0368 (2)	
C1	0.61016 (16)	0.05894 (12)	0.20951 (11)	0.0395 (3)	
C10	1.2213 (2)	0.0155 (2)	0.34728 (15)	0.0648 (5)	
C11	1.1499 (2)	0.08929 (18)	0.40684 (13)	0.0614 (5)	
C12	0.96990 (19)	0.11773 (14)	0.39499 (11)	0.0466 (4)	
C13	0.87362 (16)	0.05869 (11)	0.30592 (9)	0.0340 (3)	
C14	0.68963 (16)	0.03854 (13)	0.31557 (10)	0.0392 (3)	
C2	0.5123 (2)	0.17729 (15)	0.19553 (13)	0.0553 (4)	
C3	0.4910 (3)	0.21989 (19)	0.09124 (16)	0.0818 (7)	
C4	0.5907 (3)	0.18950 (17)	0.02523 (15)	0.0786 (7)	
C5	0.7339 (2)	0.10512 (18)	0.04581 (11)	0.0610 (5)	
C6	0.76146 (17)	0.05679 (12)	0.15111 (10)	0.0392 (3)	
C7	0.84679 (18)	−0.06886 (13)	0.16311 (11)	0.0442 (3)	
C8	0.94747 (17)	−0.05946 (12)	0.26624 (11)	0.0388 (3)	
C9	1.13154 (18)	−0.04261 (15)	0.25771 (13)	0.0531 (4)	
H1	0.9770	−0.2167	0.3197	0.074*	
H2A	0.4045	0.1650	0.2178	0.066*	
H2B	0.5683	0.2405	0.2367	0.066*	
H3	0.4022	0.2709	0.0713	0.098*	
H4	0.5705	0.2227	−0.0381	0.094*	
H5A	0.8327	0.1478	0.0313	0.073*	
H5B	0.7184	0.0360	0.0008	0.073*	
H7A	0.9182	−0.0825	0.1115	0.053*	
H7B	0.7666	−0.1346	0.1614	0.053*	
H9A	1.1803	−0.1220	0.2473	0.064*	
H9B	1.1448	0.0078	0.2002	0.064*	
H10	1.3331	−0.0010	0.3620	0.078*	
H11	1.2156	0.1257	0.4592	0.074*	
H12A	0.9561	0.2058	0.3898	0.056*	
H12B	0.9234	0.0913	0.4543	0.056*	
H14A	0.6692	−0.0438	0.3382	0.047*	
H14B	0.6492	0.0971	0.3610	0.047*	

### Atomic displacement parameters (Å^2^)

**Table:**

	*U*^11^	*U*^22^	*U*^33^	*U*^12^	*U*^13^	*U*^23^
F1	0.0442 (5)	0.0448 (5)	0.0715 (6)	−0.0124 (4)	0.0006 (4)	−0.0057 (4)
O1	0.0522 (6)	0.0300 (5)	0.0669 (7)	0.0054 (4)	0.0147 (5)	0.0108 (5)
O2	0.0422 (5)	0.0291 (5)	0.0387 (5)	−0.0068 (4)	0.0022 (4)	0.0038 (4)
C1	0.0360 (7)	0.0309 (7)	0.0508 (8)	−0.0029 (5)	0.0007 (6)	−0.0021 (6)
C2	0.0473 (8)	0.0422 (9)	0.0736 (11)	0.0093 (7)	−0.0056 (7)	−0.0028 (8)
C3	0.1079 (17)	0.0514 (11)	0.0764 (13)	0.0268 (11)	−0.0331 (12)	−0.0016 (10)
C4	0.1288 (19)	0.0463 (10)	0.0522 (10)	−0.0003 (11)	−0.0282 (11)	0.0103 (8)
C5	0.0777 (12)	0.0640 (11)	0.0400 (8)	−0.0165 (9)	0.0000 (8)	0.0061 (8)
C6	0.0445 (7)	0.0343 (7)	0.0382 (7)	−0.0062 (6)	0.0023 (6)	−0.0016 (5)
C7	0.0470 (8)	0.0374 (7)	0.0498 (8)	−0.0010 (6)	0.0127 (6)	−0.0089 (6)
C8	0.0374 (7)	0.0297 (7)	0.0503 (8)	0.0017 (5)	0.0096 (6)	0.0032 (6)
C9	0.0374 (8)	0.0498 (9)	0.0742 (11)	0.0044 (7)	0.0153 (7)	0.0081 (8)
C10	0.0351 (8)	0.0794 (13)	0.0784 (12)	−0.0069 (8)	−0.0007 (8)	0.0220 (11)
C11	0.0535 (9)	0.0697 (12)	0.0568 (10)	−0.0230 (9)	−0.0128 (8)	0.0114 (9)
C12	0.0585 (9)	0.0369 (7)	0.0423 (8)	−0.0066 (7)	−0.0036 (6)	0.0018 (6)
C13	0.0378 (7)	0.0270 (6)	0.0371 (7)	−0.0016 (5)	0.0040 (5)	0.0036 (5)
C14	0.0395 (7)	0.0354 (7)	0.0442 (7)	0.0028 (6)	0.0112 (6)	−0.0001 (6)

### Geometric parameters (Å, °)

**Table:**

F1—C1	1.4126 (15)	C7—H7A	0.9700
O1—C8	1.4272 (16)	C7—H7B	0.9700
O1—H1	0.8200	C8—C7	1.545 (2)
O2—C13	1.4650 (15)	C8—C9	1.530 (2)
O2—C6	1.4611 (16)	C9—C10	1.490 (3)
C1—C14	1.527 (2)	C9—H9A	0.9700
C1—C2	1.520 (2)	C9—H9B	0.9700
C2—C3	1.480 (3)	C10—H10	0.9300
C2—H2A	0.9700	C11—C10	1.320 (3)
C2—H2B	0.9700	C11—H11	0.9300
C3—H3	0.9300	C12—C11	1.493 (2)
C4—C3	1.316 (3)	C12—H12A	0.9700
C4—H4	0.9300	C12—H12B	0.9700
C5—C4	1.490 (3)	C13—C12	1.5109 (19)
C5—H5A	0.9700	C13—C14	1.5371 (18)
C5—H5B	0.9700	C13—C8	1.5448 (18)
C6—C1	1.538 (2)	C14—H14A	0.9700
C6—C5	1.515 (2)	C14—H14B	0.9700
C6—C7	1.5387 (19)		
			
F1—C1—C14	111.36 (11)	C6—C7—H7A	111.2
F1—C1—C2	107.49 (11)	C6—C7—H7B	111.2
F1—C1—C6	109.63 (11)	C6—O2—C13	97.25 (9)
O1—C8—C13	108.46 (11)	C8—C7—H7A	111.2
O1—C8—C7	114.30 (11)	C8—C7—H7B	111.2
O1—C8—C9	111.00 (12)	C8—C9—H9A	109.0
O2—C13—C12	112.20 (10)	C8—C9—H9B	109.0
O2—C13—C14	102.58 (10)	C8—O1—H1	109.5
O2—C13—C8	99.41 (10)	C9—C10—H10	118.4
O2—C6—C1	98.18 (10)	C9—C8—C13	110.46 (12)
O2—C6—C5	112.86 (12)	C9—C8—C7	111.63 (12)
O2—C6—C7	102.79 (11)	C10—C11—C12	123.78 (15)
C1—C14—C13	102.54 (10)	C10—C11—H11	118.1
C1—C14—H14A	111.3	C10—C9—C8	112.88 (14)
C1—C14—H14B	111.3	C10—C9—H9A	109.0
C1—C2—H2A	109.0	C10—C9—H9B	109.0
C1—C2—H2B	109.0	C11—C10—C9	123.21 (15)
C1—C6—C7	109.80 (11)	C11—C10—H10	118.4
C2—C1—C14	113.86 (12)	C11—C12—C13	114.63 (14)
C2—C1—C6	112.91 (12)	C11—C12—H12A	108.6
C2—C3—H3	118.2	C11—C12—H12B	108.6
C3—C2—C1	113.04 (15)	C12—C11—H11	118.1
C3—C2—H2A	109.0	C12—C13—C14	114.86 (12)
C3—C2—H2B	109.0	C12—C13—C8	116.47 (12)
C3—C4—C5	124.00 (17)	C13—C12—H12A	108.6
C3—C4—H4	118.0	C13—C12—H12B	108.6
C4—C3—C2	123.59 (18)	C13—C14—H14A	111.3
C4—C3—H3	118.2	C13—C14—H14B	111.3
C4—C5—C6	115.07 (16)	C13—C8—C7	100.45 (10)
C4—C5—H5A	108.5	C14—C1—C6	101.55 (10)
C4—C5—H5B	108.5	C14—C13—C8	109.40 (10)
C5—C4—H4	118.0	H12A—C12—H12B	107.6
C5—C6—C1	115.79 (13)	H14A—C14—H14B	109.2
C5—C6—C7	115.37 (13)	H2A—C2—H2B	107.8
C6—C5—H5A	108.5	H5A—C5—H5B	107.5
C6—C5—H5B	108.5	H7A—C7—H7B	109.1
C6—C7—C8	103.06 (11)	H9A—C9—H9B	107.8
			
F1—C1—C14—C13	129.67 (11)	C6—O2—C13—C14	−52.46 (11)
F1—C1—C2—C3	−78.24 (18)	C6—O2—C13—C8	59.99 (11)
O1—C8—C7—C6	127.74 (11)	C7—C6—C1—C14	61.64 (13)
O1—C8—C9—C10	−74.33 (17)	C7—C6—C1—C2	−176.04 (12)
O2—C13—C12—C11	−88.81 (15)	C7—C6—C1—F1	−56.23 (14)
O2—C13—C14—C1	23.37 (12)	C7—C6—C5—C4	152.45 (15)
O2—C13—C8—C7	−43.94 (11)	C7—C8—C9—C10	156.88 (14)
O2—C13—C8—C9	74.02 (13)	C8—C13—C12—C11	24.79 (18)
O2—C13—C8—O1	−164.13 (10)	C8—C13—C14—C1	−81.47 (12)
O2—C6—C1—C14	−45.18 (11)	C8—C9—C10—C11	−26.7 (2)
O2—C6—C1—C2	77.13 (13)	C9—C8—C7—C6	−105.25 (13)
O2—C6—C1—F1	−163.06 (10)	C12—C11—C10—C9	3.5 (3)
O2—C6—C5—C4	−89.78 (17)	C12—C13—C14—C1	145.38 (11)
O2—C6—C7—C8	24.13 (13)	C12—C13—C8—C7	−164.63 (11)
C1—C2—C3—C4	−23.8 (3)	C12—C13—C8—C9	−46.66 (16)
C1—C6—C5—C4	22.3 (2)	C12—C13—C8—O1	75.19 (14)
C1—C6—C7—C8	−79.56 (13)	C13—C12—C11—C10	−2.2 (2)
C2—C1—C14—C13	−108.60 (13)	C13—C8—C7—C6	11.85 (13)
C5—C4—C3—C2	2.2 (4)	C13—C8—C9—C10	46.01 (18)
C5—C6—C1—C14	−165.54 (13)	C13—O2—C6—C1	60.29 (11)
C5—C6—C1—C2	−43.22 (17)	C13—O2—C6—C5	−177.19 (12)
C5—C6—C1—F1	76.59 (15)	C13—O2—C6—C7	−52.26 (11)
C5—C6—C7—C8	147.40 (13)	C14—C1—C2—C3	157.92 (15)
C6—C1—C14—C13	13.05 (12)	C14—C13—C12—C11	154.56 (13)
C6—C1—C2—C3	42.79 (19)	C14—C13—C8—C7	63.06 (13)
C6—C5—C4—C3	−1.3 (3)	C14—C13—C8—C9	−178.98 (12)
C6—O2—C13—C12	−176.26 (11)	C14—C13—C8—O1	−57.12 (14)

### Hydrogen-bond geometry (Å, °)

**Table:**

*D*—H···*A*	*D*—H	H···*A*	*D*···*A*	*D*—H···*A*
O1—H1···O2^i^	0.82	2.14	2.9554 (14)	177

Symmetry codes: (i) −*x*+2, *y*−1/2, −*z*+1/2.
